# New Deoxyribozymes for the Native Ligation of RNA

**DOI:** 10.3390/molecules25163650

**Published:** 2020-08-11

**Authors:** Carolin P. M. Scheitl, Sandra Lange, Claudia Höbartner

**Affiliations:** 1 Institute of Organic Chemistry, University of Würzburg, Am Hubland, 97074 Würzburg, Germany; carolin.scheitl@uni-wuerzburg.de; 2 Agricultural Center, BASF SE, Speyerer Str 2, 67117 Limburgerhof, Germany; sandra.lange@basf.com

**Keywords:** RNA ligation, DNA catalysis, in vitro selection, deoxyribozyme

## Abstract

Deoxyribozymes (DNAzymes) are small, synthetic, single-stranded DNAs capable of catalyzing chemical reactions, including RNA ligation. Herein, we report a novel class of RNA ligase deoxyribozymes that utilize 5′-adenylated RNA (5′-AppRNA) as the donor substrate, mimicking the activated intermediates of protein-catalyzed RNA ligation. Four new DNAzymes were identified by in vitro selection from an N_40_ random DNA library and were shown to catalyze the intermolecular linear RNA-RNA ligation via the formation of a native 3′-5′-phosphodiester linkage. The catalytic activity is distinct from previously described RNA-ligating deoxyribozymes. Kinetic analyses revealed the optimal incubation conditions for high ligation yields and demonstrated a broad RNA substrate scope. Together with the smooth synthetic accessibility of 5′-adenylated RNAs, the new DNA enzymes are promising tools for the protein-free synthesis of long RNAs, for example containing precious modified nucleotides or fluorescent labels for biochemical and biophysical investigations.

## 1. Introduction

DNA is best known in the form of the famous Watson-Crick double helix of two antiparallel phosphodiester single strands held together by hydrogen bonding and stacking interactions of complementary A–T and G–C base pairs. However, the ability of DNA to form three-dimensional structures reaches far beyond the static double helix, whose main purpose is to store genetic information and faithfully transmit it to the next generation. It is now well established that the information stored in defined sequences of single-stranded DNA can be exploited for the formation of active-site architectures that catalyze chemical transformations [1,2,3]. Such “enzymes made of DNA” are known as deoxyribozymes (or DNAzymes), and they are generated in the laboratory by in vitro selection from random libraries of DNA. The first DNA enzyme reported in 1994 catalyzed the site-specific cleavage of a ribonucleotide phosphodiester bond [4]. Since then, RNA-cleaving DNA enzymes have entered various fields of fundamental and applied research [5,6,7].

**Figure 1:**
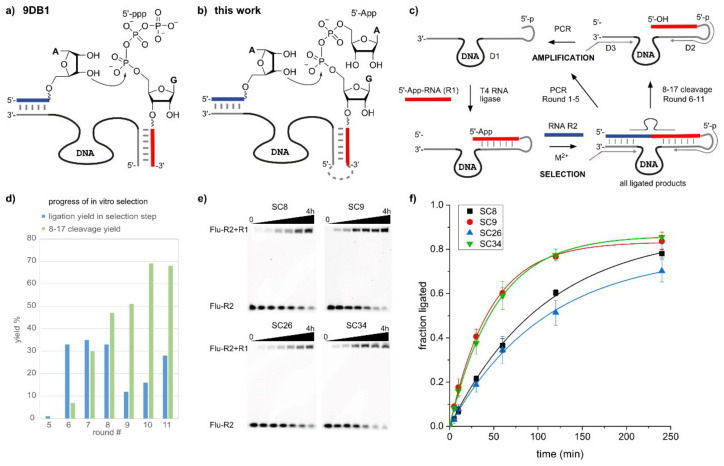
(**a**) RNA ligation reaction catalyzed by 9DB1, using a 5′-triphosphate donor RNA. (**b**) RNA ligation with 5′-adenylated (5′-App) donor RNA, for which deoxyribozymes (DNAzymes) were identified in this work. (**c**) The in vitro selection scheme involves four steps: 1. the ligation of 5′-AppRNA (R1) to the DNA library (D1); 2. the selection step, in which active DNA enzymes produce the ligation product with R2; 3. the selection pressure with 8-17, which cleaves only 3′-5′-linked RNA, but not 2′-5′ or branched RNAs; 4. PCR amplification of all active sequences (rounds 1–5) or of the 8-17-cleavable fraction (rounds 6–11). (**d**) Progress of the in vitro selection, showing ligation yield (step 2) in blue and 8-17 cleavage yield (step 3) in green. (**e**) PAGE images and (**f**) time-course of *trans*-ligation assays with individual ribozymes.

The second most important reaction catalyzed by DNA is the formation of new phosphodiester bonds for the ligation of nucleic acid fragments in different linear and branched architectures [8]. The DNA-catalyzed formation of native 3′-5′-phosphosphodiester linkages between two RNA fragments functionally mimics protein enzymes such as T4 RNA ligase and facilitates protein-free access to large synthetic RNAs [9,10]. This linear RNA ligation requires the regioselective activation of the 3′-hydroxy group of one RNA fragment (the acceptor RNA) for the nucleophilic attack at an activated 5′-phosphate group of the second RNA fragment (the donor RNA). The leading DNA catalyst for this type of reaction is the 9DB1 deoxyribozyme [9], which uses a 5′-triphosphate as the electrophile (Figure 1a). Similar to the majority of DNA enzymes, the 9DB1 DNA enzyme hybridizes to its two RNA substrates via Watson-Crick base pairing. The reactive termini are arranged in the active site such that only the 3′-OH group can serve as a nucleophile. The formation of native linkages was enforced during the in vitro selection strategy devised by Silverman [9], by allowing only DNAzymes linked to 3′-5′-ligated products to be amplified in subsequent selection rounds.

The catalytic activity of RNA-ligating DNA enzymes may also be compared to ligase and polymerase ribozymes that are discussed in the context of the RNA world hypothesis [11,12]. Moreover, the chemical reaction catalyzed by the 9DB1 ligase mimics the elongation step of RNA by polymerases, which use nucleotide triphosphates as substrates. As for natural polymerases, the only cofactor necessary for 9DB1 is Mg^2+^ as a divalent metal ion. The 9DB1 DNA enzyme was heavily investigated biochemically [13,14], and it was the first DNA catalyst for which a three-dimensional structure was determined by X-ray crystallography [15]. The structure revealed a 31-nt double-pseudoknot architecture in the catalytic core containing long-range Watson–Crick base pairs and non-canonical hydrogen-bonding interactions that could not be predicted from any biochemical data. The structure also explained the regioselectivity of ligation and proposed the involvement of a critical phosphodiester of the DNA backbone in the activation mechanism, which was further investigated by computational analyses [16,17,18].

An important aspect for practical applications of DNA enzymes is their substrate preference in terms of accepted RNA sequences and the accessibility of donor substrates with activated phosphate groups [10,19]. The 9DB1 deoxyribozyme preferably ligates RNA substrates with 5′-triphosphorylated guanines on the donor strand, and the crystal structure revealed the reason for the sequence preferences [15]. Structure-guided mutagenesis of the DNA enzyme enabled the engineering of mutant deoxyribozymes that allow the ligation of previously inactive substrate sequences, such as RNAs with 5′-triphosphorylated pyrimidines at the 5′-end [9,15]. However, such RNA substrates are not accessible by in vitro transcription with T7 RNA polymerase (which requires GTP for initiation), and instead, they need to be synthesized chemically. Advanced methods for 5′-triphosphorylation by solid-phase synthesis have been reported to facilitate access to these desired ligation substrates [20,21], but the custom synthesis of 5′-pppRNA containing modified nucleotides is not yet routinely available. An RNA-catalyzed synthesis of 5′-triphosphates may also be considered, but the accessible substrate scope is limited [22]. Therefore, we asked the question of whether the 9DB1 DNA enzyme would accept alternatively activated phosphate electrophiles as donor substrates, and we considered 5′-adenylated RNA (5′-AppRNA) as a potential candidate (Figure 1b). 5′-AppRNA is the active intermediate during RNA ligation by protein enzymes (such as T4 RNA ligase), which activate the 5′-phosphate of the RNA donor by adenylation with ATP, and upon reaction with the 3′-OH of the acceptor RNA, AMP is released as a leaving group [23]. A number of convenient routes for the synthesis of 5′-adenylated oligonucleotides have been reported [24,25,26], and these were previously used as donor substrates for in vitro selections of catalytic DNA. Several deoxyribozymes are known to form 2′,5′-branched nucleic acid architectures with 5′-adenylated RNA and DNA [27,28]. However, when the 9DB1 DNA enzyme was tested with 5′-AppRNA instead of 5′-pppRNA, only poor ligation yields were obtained (Figure S1, <10% compared to >85% with 5′-pppRNA). As of now, there was no deoxyribozyme ligase available for the linear native ligation of 5′-AppRNA fragments.

In this work, we report new RNA ligase deoxyribozymes that enable native linear RNA ligation using 5′-AppRNA as a donor substrate. Using in vitro selection, four new DNA catalysts were identified and biochemically characterized. With MnCl_2_ as a cofactor, the SC9 DNA enzyme enables general RNA ligation in good yields (ca 80% ligation yield in 4 h). Future biochemical and structural investigations of the new DNA enzymes are promising to reveal more detailed insights into fundamental aspects of DNA catalysis in comparison to ribozymes and protein enzymes.

## 2. Results and Discussion

### 2.1. In Vitro Selection of RNA-Ligating Deoxyribozymes

DNA enzymes with catalytic activity for the desired RNA ligation were identified by in vitro selection from a DNA library containing 40 random nucleotides (Figure 1c), following a similar strategy as previously reported for the identification of 9DB1 [9]. In the present work, a 5′-adenylated RNA selection substrate was prepared from the synthetic 5′-phosphorylated RNA oligonucleotide R1 by incubation with AMP-imidazolide and MgCl_2_, as previously described [24,29]. Then, the 5′-AppRNA was covalently ligated to the 5′-phosphorylated synthetic DNA library (D1) using T4 RNA ligase. The key incubation step during in vitro selection was performed with 5′-^32^P-labeled RNA R2 in the presence of 20 mM MgCl_2_ and 20 mM MnCl_2_ at pH 7.5. The ligated active fraction of the DNA library was separated by PAGE and amplified by PCR with a 5′-phosphorylated forward primer (D2) and a tailed reverse primer (D3). The asymmetric double-stranded PCR product was separated into single strands by PAGE, and the shorter 5′-phosphorylated strand representing the enriched DNA library was again ligated to RNA substrate R1 to initiate the next round of selection. The incubation time in rounds 1–7 was 15 h, which was reduced to 5 h in round 8, and 1 h in rounds 9–11. The fraction of ligation product in each round was quantified (Figure 1d), and as soon as a significant amount of ligation product was detectable in round 5, an additional step was included in every selection round to enforce the enrichment of 3′-5′-ligated products. Therefore, the isolated ligation product was treated with an 8-17 deoxyribozyme (D4) targeted toward the ligation site, which is known to cleave only 3′-5′ linked RNA, and the cleaved fraction was isolated and amplified. In round 11, the activity reached 28% ligation product, of which 68% was cleavable by 8-17. Individual DNA catalysts were identified from the round 11 DNA library by TOPA-TA cloning and Sanger sequencing of 15 isolated plasmids. Four new DNA enzymes were identified and named according to the arbitrary clone number (Table 1). The dot plots and predicted minimum free energy secondary structures are depicted in Supplementary Figure S2.

The DNA enzymes were independently synthesized by solid-phase synthesis and tested for catalytic activity in *trans*. Each of the DNA enzymes SC8, SC9, SC26, and SC34 was annealed with R1 and 5′-fluorescein-labeled R2, and the reaction mixture was incubated with 40 mM MnCl_2_ at pH 7.5. The reactions were run in triplicate and analyzed by denaturing PAGE (Figure 1e), and *k*_obs_ was determined by fitting the yield versus time data to a pseudo-first order kinetic model (Figure 1f). The *k*_obs_ values are listed in Table 1 and demonstrate that SC9 and SC34 were about two-fold faster than SC8 and SC26. Since SC9 was the most abundant and fastest DNA enzyme among the isolated clones, it was chosen for further characterization.

### 2.2. The SC DNA Enzymes Synthesize Native 3′-5′-Linked RNA

**Figure 2:**
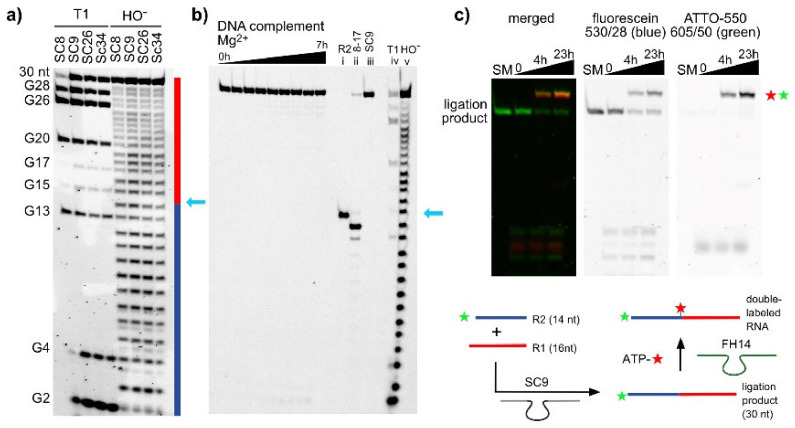
(**a**) The isolated 30-nt ligation products generated by SC8, SC9, SC26, and SC34 were analyzed by alkaline hydrolysis and RNase T1 digestion. The results confirm the continuous backbone of the linear RNA; the light blue arrow indicates the ligation site. (**b**) The ligation product of SC9 was hybridized to the complementary DNA (D5) and incubated with 100 mM MgCl_2_ at pH 9.0 for up to 7 h. No cleavage was observed. In addition, site-specific cleavage by the 8-17 deoxyribozyme (lane ii, 20 mM Mg^2+^, 20 mM Mn^2+^, 40 mM Tris-HCl, pH 7.5, 5 h) confirms the 3′-5′-linkage at the ligation site (see Supplementary Figure S3 for additional 8-17 cleavage results). (**c**) The SC9 ligation product was used for FH14-catalyzed RNA labeling with Atto550-ATP (200 µM; 40 mM MgCl_2_, 50 mM HEPES, pH 7.5, 37 °C, up to 23 h). Formation of double-labeled product confirmed the native RNA constitution.

To confirm the linear and native nature of the ligated RNAs, the ligation products generated by each of the four deoxyribozymes were isolated and subjected to alkaline hydrolysis. A continuous single-nucleotide resolution ladder was obtained, and no missing or shifted bands were observed (Figure 2a). These results confirmed the linear constitution of the product, since all internal ribose 2′-OH groups were freely available for attack at the adjacent phosphodiester bond. Moreover, upon incubation with the 8-17 deoxyribozyme, the RNA strand was cleaved at the desired site (Figure 2b and Supplementary Figure S3). The cleavage product lined up with the corresponding band in the alkaline hydrolysis lane, but it was shifted with respect to the reference RNA R2, because the cleavage product contains a 2′,3′-cyclic phosphate which is known to migrate faster on PAGE. The ligation product was hybridized to the complementary DNA strand (D5) and incubated with Mg^2+^ to reveal possible 2′-5′-linked products, which are known to be less stable under these incubation conditions compared to 3′-5′-linked products [30]. No cleavage products were observed, further supporting the desired identity of the RNA. As an additional confirmation, we checked if the ligation product could serve as a substrate for RNA-catalyzed labeling at the ligation junction. The recently reported FH14 ribozyme uses the 2′-OH group of an internal adenosine for labeling with fluorescent *N*^6^-aminohexyl-ATP via the formation of a 2′,5′-branched phosphodiester linkage [31,32]. Fluorescein-labeled R2 was ligated with R1 by incubation with SC9, and the resulting product was then treated with FH14 and Atto550-ATP. Figure 2c shows the formation of the Atto550- and fluorescein- double-labeled RNA product.

### 2.3. The SC9 Deoxyribozyme Requires Mn^2+^ as a Cofactor

**Figure 3:**
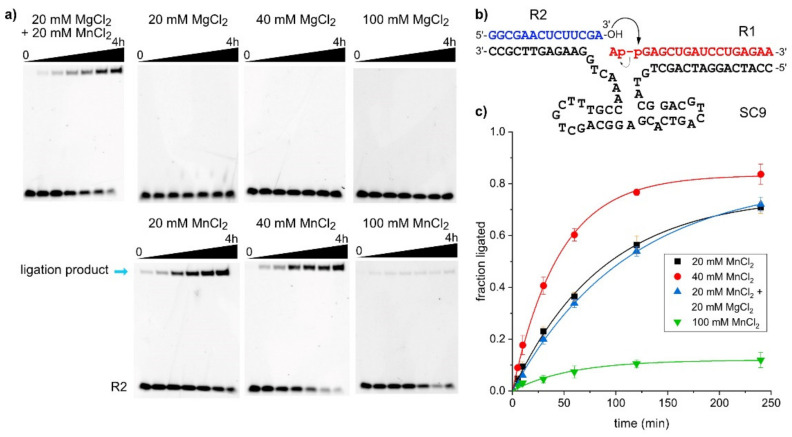
Metal ion requirement of SC9. (**a**) PAGE images of kinetic experiments under different metal ion conditions. (**b**) Predicted secondary structure scheme of RNA substrates hybridized to SC9 deoxyribozyme used in these experiments. (**c**) Yield versus time data from three independent experiments, represented as mean ± stdev and fitted to pseudo-first order kinetics. *k*_obs_ values are 0.012 (20 mM Mn^2+^), 0.022 (40 mM Mn^2+^), 0.009 (20 mM Mn^2+^ + 20 mM Mg^2+^); final yield is only 12% for 100 mM Mn^2+^.

The majority of deoxyribozymes require divalent metal ions as cofactors. Exceptions are Na^+^-specific DNAzymes [33,34,35,36], and several examples carrying amino-acid-like functional groups on modified nucleotides [37,38], to catalyze transesterification reactions resulting in strand cleavage. The large class of 8-17 deoxyribozymes that collectively cleave the majority of dinucleotide junctions when a single ribonucleotide is embedded in a DNA backbone [39] perform best with equimolar mixtures of Mg^2+^ and Mn^2+^, and they are usually employed at 10–20 mM each [5]. All known RNA ligase deoxyribozymes, including 9DB1, use M^2+^ cofactors (ligase deoxyribozymes using modified nucleosides have not yet been reported). In vitro selection of the 9DB1 RNA ligase was carried out at pH 9.0, and the deoxyribozyme was trained to use Mg^2+^ [9]. Later, it was demonstrated that 9DB1-catalyzed RNA ligation also occurred at pH 7.5. The reaction rate was significantly reduced at this more RNA-friendly pH with Mg^2+^ as a cofactor, but faster reaction rates were restored when Mg^2+^ was replaced by Mn^2+^ [10]. Capitalizing on the reported advantages of Mn^2+^ for DNA catalysis, the in vitro selection in this work was carried out at pH 7.5 in the presence of 20 mM each of MnCl_2_ and MgCl_2_, and the metal ion dependence of the SC9 deoxyribozyme was determined (similar results were obtained for the other three DNA enzymes). The ligation reaction was carried out under different conditions, and the amount of ligation product at various time points was determined by PAGE. Representative gel images and the kinetic graphs for the seven tested conditions are depicted in Figure 3. Interestingly, the SC9 deoxyribozyme requires the presence of Mn^2+^ in the ligation buffer, and it is inactive when only Mg^2+^ is supplied as a divalent metal ion cofactor, up to a concentration of 100 mM. Doubling the concentration of Mn^2+^ ions from 20 to 40 mM resulted in about two-fold faster reaction, while a further increase to 100 mM Mn^2+^ dramatically reduced the ligation yield. The surprising finding that Mg^2+^ does not play a productive role in SC9 catalysis is supported by the observation that the reaction rates at 20 mM Mn^2+^ and a mixture of 20 mM Mn^2+^ + 20 mM Mg^2+^ are nearly indistinguishable.

### 2.4. The SC9 Deoxyribozyme Has a Broad RNA Substrate Scope

**Figure 4:**
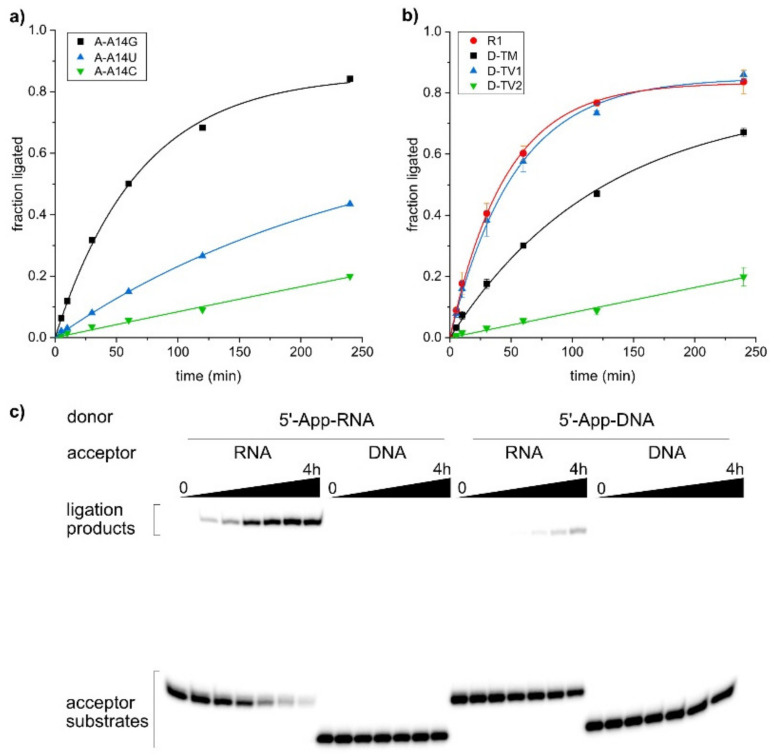
SC9-catalyzed ligation of mutated substrates. (**a**) Kinetic graphs of ligation with mutations of the 3′-terminal nucleotide in the acceptor RNA. (**b**) Kinetic graphs for ligation of transition and transversion mutants of donor RNA (5′-AppG was kept as in parent RNA). See Table 2 for sequences of RNA substrates and corresponding SC9 deoxyribozyme mutants. (**c**) Gel images of ligation tests with DNA/RNA substrate combinations.

RNA-ligating deoxyribozymes are useful tools for the synthesis of site-specifically labeled RNA [9,10]. A prerequisite for general application is a broad substrate scope, enabling the efficient ligation of diverse RNA substrate sequences. Here, we tested the ability of the SC9 deoxyribozyme to ligate transition and transversion mutations of the original selection substrate. For every change in the RNA substrate, the binding arms of the deoxyribozyme were also varied to maintain Watson-Crick complementarity. The 5′-App donor RNAs and the fluorescein-labeled acceptor RNAs were incubated with the corresponding deoxyribozyme in the presence of 40 mM MnCl_2_ at pH 7.5, and the ligation yield at various time points was determined by PAGE. The yield versus time data are plotted in Figure 4a,b.

First, we checked the variability of the 3′-terminal nucleotide of the acceptor RNA. The best ligation yields were obtained when the acceptor substrate terminated with a purine nucleotide, and slightly less but still useful yields were obtained with pyrimidines (G~A > U > C). On the other hand, only 5′-AppG was accepted at the 5′-end of the donor substrate, while all nucleotides beyond this first position in the donor RNA permitted the tested mutations. The collective changes in the binding arm to transversions-1 (G↔C, A↔U) were very well tolerated, resulting in equally fast and efficient ligation as the parent substrate. The ligation rate and yield were only slightly reduced for transition mutations (G↔A, U↔C), while the RNAs generated by transversion-2 (G↔U, A↔C) mutations turned out as poorer substrates, resulting in only 20% ligated product after 4 h incubation time.

These results are comparable to the originally reported substrate scope of the 9DB1 DNA enzyme [9], which was substantially expanded once the three-dimensional structure was known [15]. The crystal structure of 9DB1 in complex with the ligation product revealed the key recognition nucleotides for the nucleotides at the ligation junction, and structure-guided compensatory mutations in the catalytic core enabled the synthesis of engineered deoxyribozymes for the ligation of all dinucleotide junctions. It may not be unreasonable to expect that similar structure-guided mutations could lead to an expanded substrate scope of the SC9 deoxyribozyme.

The second aspect of the substrate scope beyond the nucleotide sequence addresses the backbone constitution. A fundamental question asks whether deoxyribozymes trained to ligate RNA may be able to tolerate the removal of the 2′-OH group in the substrate, i.e., if DNA oligonucleotides can be used as substrates. It is interesting to note that deoxyribozymes that form 2′,5′-branched nucleic acid architectures have been developed for all four combinations of DNA and RNA substrates, i.e., both the scaffold and adaptor strands can be either DNA or RNA (for a summary see [40]). Although dedicated deoxyribozymes for all combinations have been identified by in vitro selection [27,28,41,42], some DNA enzymes show substrate promiscuity and are able to ligate more than one type of polynucleotide. For example, the 10DM24 and 9FQ4 deoxyribozymes accept 5′-AppRNA and 5′-AppDNA as a substrate for ligation to the 2′-OH of the adenosine branch site in the target RNA [43] (see also Supplementary Figure S4). Here, we addressed the question of whether the new SC9 deoxyribozyme would also be able to generate RNA-DNA hybrid structures. In particular, the ligation of a DNA donor to an RNA acceptor could have practical utility for the attachment of primer binding sites for RNA deep sequencing applications. Therefore, we synthesized the corresponding DNA donor and acceptor substrates and examined the ability of SC9 to ligate all four combinations. The results are depicted in Figure 4c and reveal significantly reduced activity with 5′-AppDNA as compared to 5′-AppRNA as a donor. DNA is not permitted as the acceptor substrate, and the ligation of two DNA substrates cannot be achieved with the SC9 deoxyribozyme. These results demonstrate the high specificity of the new Mn^2+^-dependent DNA catalyst for RNA ligation substrates. The residual activity with the 5′-AppDNA donor encourages further optimization of the catalyst, which is likely achievable by few rounds of in vitro evolution from a partially randomized SC9 library.

## 3. Conclusions

In this work, we reported a new class of DNA catalysts for RNA ligation using 5′-adenylated donor RNA as a substrate. For synthetic RNAs that contain precious modified nucleosides, activation of the 5′-phosphate as 5′-adenylate is synthetically more easily accessible than 5′-triphosphorylation. Therefore, the SC9 and related deoxyribozymes will likely find application as synthetic tools for RNA ligation. The new catalysts currently ligate 5′-AppG RNAs most efficiently, but mutations beyond the first nucleotide are well tolerated as long as Watson–Crick complementarity to the binding arm is maintained. The SC9 deoxyribozyme can only use Mn^2+^ ions as a cofactor, while smaller Mg^2+^ ions are not suitable to support the catalysis of RNA ligation. It will be interesting to investigate the metal-ion dependent folding of the deoxyribozyme to identify key structural differences that are responsible for the metal ion preference [44]. In the future, we will use combinatorial mutagenesis [14,45] and nucleotide analog interference mapping of DNA [13] to guide the minimization of the catalytic core. From screening crystallization constructs with variations in the lengths of the substrate binding arms and subsequent structural analyses, we expect new insights into the organization of the catalytic core that accommodates the significantly larger AMP leaving group compared to the pyrophosphate leaving group in 9DB1 [15]. In the best-case scenario, crystal structure analyses may help disclose a recognition nucleotide for the adenylated 5′-guanosine and suggest compensatory mutations to further expand the substrate scope of SC9. Additional future directions consider the evolution of bifunctional nucleic acid catalysts that may catalyze the activation and the ligation steps (for example, by using ATP as cofactor [46]), or that use alternative activated donor substrates, such as phosphor-2-aminoimidazolides that were recently explored as prebiotically plausible substrates for RNA-ligating ribozymes [47].

## 4. Materials and Methods

### 4.1. General Information and Sequences of DNAs and RNAs Used in This Study

DNA oligonucleotides were purchased from Microsynth and purified by denaturing PAGE prior to use. RNA oligonucleotides were prepared by solid-phase synthesis using phosphoramidite chemistry (2′-*O*-TOM-protected) on CPG solid supports as previously reported [48], which were deprotected with ammonia/methyl amine, followed by 1 M tetrabutylammonium fluoride in THF, desalted, and purified by denaturing polyacrylamide gel electrophoresis (PAGE). The sequences of all synthetic DNA and RNA oligonucleotides used in this work are listed in Table 2. The quality of RNAs (purity and identity) was analyzed by Anion exchange HPLC (Dionex DNAPac PA200, 2 × 250 mm at 60 °C. Solvent A: 25 mM Tris-HCl (pH 8.0), 6 M Urea. Solvent B: 25 mM Tris-HCl (pH 8.0), 6 M Urea, 0.5 M NaClO_4_. Gradient: linear, 0–40% solvent B, 4% solvent B per 1 CV) and HR-ESI-MS (micrOTOF-Q III, negative mode, direct injection). Solid supports and phosphoramidite building blocks for solid-phase synthesis were purchased from ChemGenes. 6-FAM-azide, *N*^6^-(6-Amino)hexyl-ATP-ATTO-550, deoxyribo- and ribonucleotide triphosphates (dNTPs and NTPs) were purchased from Jena Bioscience. T4 Polynucleotide Kinase (PNK), RNase T1, T4 DNA Ligase, T4 RNA Ligase, and DreamTaq DNA polymerase were purchased from Thermo Fisher Scientific. T7 RNA polymerase was prepared in house following a published procedure with minor modifications [49]. γ-^32^P-ATP and α-^32^P-dATP were from Hartmann Analytic GmbH. Fluorescence gel images were recorded using a ChemiDoc MP imager with epi illumination using blue and green LEDs (emission filters 530/28 (blue), 605/50 (green)) from Biorad. Radioactive gels were dried under vacuum, exposed to phosphor storage screens, and imaged using a Typhoon Phosphorimager from GE Healthcare.

### 4.2. Synthesis of Donor and Acceptor Substrates

Acceptor substrates were synthesized as 5′-hexynyl RNA and labeled with fluorescein using CuAAC (click) chemistry or synthesized with free 5′-OH and labeled with ^32^P. First, 5 nmol of synthetic 5′-alkyne functionalized oligonucleotides were dissolved in 10 µL of a freshly prepared solution of H_2_O/DMSO/*t*BuOH (4:3:1) containing 5 mM 5-FAM-azide, 5 mM CuBr, and 10 mM TBTA. After incubation at 37 °C for 3 h in the dark, the labeled oligonucleotides were precipitated and purified by PAGE. Radiolabeling reaction was performed in a total volume of 10 µL using 100 pmol of oligonucleotide carrying a free 5′-OH group. The reactions were incubated at 37 °C for 1 h in 1× PNK buffer A (50 mM Tris, pH 7.6, 10 mM MgCl_2_, 5 mM DTT, 0.1 mM spermidine) in the presence of 5 µCi γ-^32^P-ATP and 5 U of T4 PNK. The labeled product was purified by PAGE.

5′-adenylated donor substrates were prepared in two steps. First, 4 nmol of oligonucleotides with free 5′-OH were 5′ phosphorylated in 20 µL of 1× T4 PNK buffer A (50 mM Tris, pH 7.6, 10 mM MgCl_2_, 5 mM DTT, 0.1 mM spermidine) in the presence of 1 mM ATP using 10 U of T4 PNK. After incubation at 37 °C for 4 h, the oligonucleotides were precipitated and used for the 5′-adenylation reaction without further purification. Then, 30 µL of a solution containing 100 mM AMP-imidazolide (prepared according to [24]) and 50 mM MgCl_2_ were added to the dry 5′-phosphorylated oligonucleotides. After incubation at 50 °C for 1 h, another 15 µL of the 100 mM AmpI/50 mM MgCl_2_ solution was added followed by incubation for another 2 h at 50 °C. The RNA was precipitated and purified on denaturing PAGE yielding 45–55% 5′-adenylated product. The identity of the adenylated product was confirmed by ESI-MS mass spectrometry (see Supplementary Materials Table S1).

### 4.3. In Vitro Selection

#### 4.3.1. Preparation of the Starting Pool

First, 1.5 nmol 5′-phosphorylated N40-DNA-pool (D1) was annealed to 1.8 nmol donor RNA (R1) (95 °C for 3 min, 10 min at 25 °C), and 1 mM ATP, T4 RNA ligase (0.5 U/μL), 50 mM Tris-HCl pH 7.5, after which 10 mM MgCl_2_ and 10 mM DTT were added to yield a total reaction volume of 30 µL. After 6 h incubation at 37 °C, the ligation product was purified by PAGE.

The ^32^P-labeled size standard was generated by two ligation reactions. First, R2 (40 µM) and R1 (40 µM) were ligated by T4 DNA-ligase using a DNA-splint (D5; 40 µM). After annealing (95 °C for 3 min, 10 min at 25 °C) of the three oligonucleotides, 40 mM Tris-HCl pH 7.8, 10 mM MgCl_2_, 10 mM DTT, 5 mM ATP, and 0.5 U/µL T4 DNA ligase were added. The reaction mixture was incubated at 37 °C for 16 h and the ligation product was purified by PAGE. Second, the RNA thus generated was ligated to 5′-^32^P-phosphorylated D1 by T4 RNA-ligase as described above.

#### 4.3.2. Selection Step

For the first selection round, 50 pmol ^32^P-labeled and 450 pmol unlabeled D1-R1 ligation product were annealed (95 °C for 3 min, 10 min at 25 °C) with 1 nmol R2 in 28 μL 1× Annealing buffer (25 mM HEPES pH 7.5, 0.1 mM EDTA, 15 mM NaCl). Then, 8 µL 5× reaction buffer (1× concentration: 50 mM HEPES pH 7.5, 2 mM KCl, 150 mM NaCl) were added, and the reaction was started by the addition of 20 mM MgCl_2_ and 20 mM MnCl_2_ to yield a final reaction volume of 40 µL. The reaction mixture was incubated overnight (15 h) at 37 °C, followed by PAGE purification, in which the band next to the size standard was excised and extracted. The reaction time was reduced to 5 h in round 8, and 1 h from round 9 onwards.

To favor the enrichment of DNAzymes catalyzing the formation of native 3′-5′-phosphodiester linkages, an additional selection pressure was introduced starting from round 6. The PAGE-purified ligation products were cleaved by the 8-17 deoxyribozyme (D4), upon incubation with 10 µM 8-17 deoxyribozyme (D4) in 40 mM Tris-HCl, pH 7.5, 150 mM NaCl, 20 mM MgCl_2_, and 20 mM MnCl_2_ at 37 °C. After 4 h incubation, the cleavage product was purified by PAGE and submitted to PCR amplification.

#### 4.3.3. PCR Amplification of the Enriched Pool

For amplification of the enriched pool, the isolated oligonucleotides were mixed with 2 µM 5′-phosphorylated forward primer (D2), 0.5 µM reverse primer (D3), 0.2 mM dNTPs, and 5 U Taq DNA-polymerase in the presence of 1× Taq-buffer (10 mM Tris-HCl, 50 mM KCl, 1.5 mM MgCl_2_, pH 8.3). The final reaction volume was 100 µL. Amplification was performed under the following conditions: 94 °C/5 min, 94 °C/30 s, 48.3 °C/30 s, 72 °C/30 s with 9 additional cycles from step two and final 72 °C/4 min.

After phenol/chloroform/isoamyl alcohol (PCI) extraction, 1 µL of the 10-cycle PCR product was used as input for a 30-cycle PCR reaction (40 µL), using 0.2 mM of a dNTP mixture containing α-^32^P-dATP, 2.5 µM 5′-phosphorylated forward primer (D2), and 625 nM reverse primer (D3). All other reaction conditions remained identical to the first PCR amplification.

The asymmetric double-stranded PCR product was separated into single strands by PAGE. The shorter strand, representing the enriched, 5′-phosphorylated DNA-library, was extracted and annealed (95 °C for 3 min, 10 min at 25 °C) with 10 µM donor RNA R1. Then, 1 mM ATP, T4 RNA-Ligase (0.5 U/μL), 50 mM Tris-HCl pH 7.5, 10 mM MgCl_2_, and 10 mM DTT were added, and the reaction mixture was incubated at 37 °C for 3 h. The ligation product was purified on PAGE and submitted to the next selection round.

#### 4.3.4. Cloning and Sequencing of the Enriched Pool

After 11 rounds of selection, the enriched library was cloned using TOPO-TA cloning and transformation into *E. coli*. The plasmids of 40 randomly picked colonies were isolated and tested for activity. The 15 most active clones were sequenced using Sanger sequencing, and the results are listed in Table 1.

### 4.4. Characterization of the Deoxyriboyzmes

#### 4.4.1. Kinetic Analysis of Intermolecular Ligation Activity (Reaction in *trans*)

In 7 μL 1× annealing buffer (25 mM HEPES pH 7.5, 0.1 mM EDTA, 15 mM NaCl), 10 pmol 5′-flourescein or ^32^P-labeled acceptor oligonucleotide were annealed (95 °C for 3 min, 10 min at 25 °C) with 60 pmol 5′-adenylated donor oligonucleotide and 40 pmol DNAzyme. Then, 2 µL 5× reaction buffer (1× concentration: 50 mM HEPES pH 7.5, 2 mM KCl, 150 mM NaCl) were added, and the reaction was started by addition of the respective divalent metal ion stock solution to a final reaction volume of 10 µL (to give a final concentration of 20, 40, or 100 mM MnCl_2_ or MgCl_2_, or a mixture of both at 20 mM each). The reactions were incubated at 37 °C, after which 1 µL aliquots were taken after certain time points, and the reaction was quenched immediately by the addition of 4 μL of stop solution and freezing. Half of each time point sample was analyzed by denaturing PAGE, and band intensities were quantified by phosphor imaging or by fluorescence imaging using blue epi illumination and 530/28 nm emission filter. The yield versus time data were fit to (fraction ligated) = Y*(1 − e^−kt^), where k = *k*_obs_ and Y = max final yield.

#### 4.4.2. Characterization of the Ligation Products

The ligation products were prepared by annealing 200 pmol of each RNA (R1 and R2) with 200 pmol of the respective DNAzyme in 14 μL 1× annealing buffer (25 mM HEPES pH 7.5, 0.1 mM EDTA, 15 mM NaCl, 95 °C for 3 min, 10 min at 25 °C). Then, 4 µL of 5× reaction buffer (1× conc.: 50 mM HEPES pH 7.5, 2 mM KCl, 150 mM NaCl) were added, and the reaction was started by addition of 40 mM MnCl_2_ to yield a final reaction volume of 20 µL. After overnight incubation (22 h) at 37 °C, the ligation product was purified on denaturing PAGE and used for the following experiments.

##### RNase T1 Digestion

In 5 µL of 50 mM Tris-HCl (pH 7.5), 5 pmol ligated RNA was digested with 0.5 U RNase T1 for 30 s at 37 °C. The reaction was quenched by the addition of 7.5 μL stop solution and placing the reaction mixtures on ice.

##### Alkaline Hydrolysis

First, 5 pmol ligation product was heated at 95 °C in 5 µL 50 mM NaOH. After 2.5 min, the reaction was quenched by adding 7.5 μL stop solution and placing the sample on ice. All samples were resolved on denaturing PAGE and then subjected to fluorescence imaging using blue epi illumination and a 530/28 nm emission filter.

##### Cleavage by 8-17 Deoxyribozyme

First, 5 pmol ligation product was annealed with 50 pmol 8-17 deoxyriboyzme (D4); then, it was incubated with 20 mM MgCl_2_ and 20 mM MnCl_2_ in 40 mM Tris-HCl, pH 7.5, 150 mM NaCl in a final volume of 5 µL. The reaction mixture was incubated at 37 °C for 5 h, quenched with stop solution, and analyzed by PAGE next to an alkaline hydrolysis and T1 digestion ladder.

##### MgCl_2_ Cleavage in RNA-DNA Duplex

First, 10 pmol ligation product was annealed with 100 pmol complementary DNA (D5) and incubated with 100 mM MgCl_2_, in 50 mM CHES buffer, pH 9.0, 150 mM NaCl, 2 mM KCl, in a final volume of 10 µL at 37 °C for up to 7 h. Then, 1 µL aliquots were removed at 30 min and then at every hour, mixed with 4 µL stop solution, and analyzed by PAGE.

##### In Situ Labeling of the Ligation Product by FH14 Ribozyme Using *N*^6^-(6-amino)hexyl-ATP

For RNA ligation, 40 pmol of DNAzyme SC9, 10 pmol 5′-fluorescein-labeled R2, and 60 pmol R1 were annealed (95 °C for 3 min, 10 min at 25 °C) in 7 μL 1× annealing buffer (25 mM HEPES pH 7.5, 0.1 mM EDTA, 15 mM NaCl). Then, 2 µL of 5× reaction buffer (1× conc.: 50 mM HEPES pH 7.5, 2 mM KCl, 150 mM NaCl) were added, and the reaction was started by the addition of 40 mM MnCl_2_ to yield a final reaction volume of 10 µL. After overnight incubation (22 h) at 37 °C, the reaction mixture was precipitated and submitted to the labeling reaction without further purification.

Half of the ligation sample was mixed with 60 pmol of the corresponding FH14 ribozyme in 5 μL of ribozyme reaction buffer (120 mM KCl, 5 mM NaCl, 50 mM HEPES, pH 7.5), including 200 µM of *N*^6^-(6-amino)hexyl-ATP-ATTO-550 and 40 mM MgCl_2_. An annealing step (3 min at 95 °C, 10 min at 25 °C) was performed prior to the addition of MgCl_2_ and the ATP substrate. The reaction mixture was incubated at 37 °C. Then, 1 µL aliquots were taken after certain time points, and the reaction was quenched immediately by the addition of 99 µL TEN buffer. All time point samples were precipitated and dissolved in 5 µL stop solution. Half of each time point sample was analyzed by denaturing PAGE, and band intensities were quantified by fluorescence imaging using blue epi illumination (530/28 nm emission filter) and green epi illumination (605/50 nm emission filter).

## Figures and Tables

**Table 1:** Overview of deoxyribozyme core sequences and observed rate constants.

DNAzyme	Core Sequence	Number of Clones	*k*_obs_ (min^−1^) ^1^
**SC8**	GCGGGCCCCAATTCATTGGCTTAACTACGAGGACGTCCAG	1	0.0092 ± 0.0004
**SC9**	GTACGGACGTCAGTCACGAGGCAGCTGCTTTGCCAAACTG	8	0.0219 ± 0.0006
**SC26**	ACCGTCACAAAAGACTGTAGTTAATACCACTGCAGGTCGT	5	0.0093 ± 0.0004
**SC34**	GCACAGGGGTATAATTGCCTCGTACAACTTTATGTCCGAA	1	0.0193 ± 0.0003

^1^ measured under single-turnover conditions with 40 mM MnCl_2_, in 50 mM HEPES, pH 7.5.

**Table 2:** Names and sequences of synthetic DNAs and RNA oligonucleotides.

Name	5′-Sequence-3′
D1 DNA Pool	pCCATCAGGATCAGCT-N40-GAAGAGTTCGCCGp
D2 Fwd primer	pCCATCAGGATCAGCT
D3 Rev primer	ACCACCAACAACA-X(spacer18)-ATGCTCGGCGAACTCTTC
D4 8-17	GATGGTTCAGGATCAGCTTCCGAGCCGGACGACGAAGAGTTCGCC
D5 Splint	CAGGATCAGCTCTCGAAGAGTTC
SC8	CCATCAGGATCAGCTGCGGGCCCCAATTCATTGGCTTAACTACGAGGACGTCCAGGAAGAGTTCGCC
SC9	CCATCAGGATCAGCTGTACGGACGTCAGTCACGAGGCAGCTGCTTTGCCAAACTGGAAGAGTTCGCC
SC26	CCATCAGGATCAGCTACCGTCACAAAAGACTGTAGTTAATACCACTGCAGGTCGTGAAGAGTTCGCC
SC34	CCATCAGGATCAGCTGCACAGGGGTATAATTGCCTCGTACAACTTTATGTCCGAAGAAGAGTTCGCC
SC9-D-TM	CCACTGAAGCTGATCGTACGGACGTCAGTCACGAGGCAGCTGCTTTGCCAAACTGGAAGAGTTCGCC
SC9-D-TV1	CCAAGTCCTAGTCGAGTACGGACGTCAGTCACGAGGCAGCTGCTTTGCCAAACTGGAAGAGTTCGCC
SC9-D-TV2	CCAGACTTCGACTAGGTACGGACGTCAGTCACGAGGCAGCTGCTTTGCCAAACTGGAAGAGTTCGCC
9DB1	CAGGATCAGCTGGATCATACGGTCGGAGGGGTTTGCCGTTTACGAAGAGTTCGC
Donor DNA	GAGCTGATCCTGAGAA
Acceptor DNA	GGCGAACTCTTCGA
R1 Donor	GAGCUGAUCCUGAGAA
R2 Acceptor	GGCGAACUCUUCGA
D-TM	G*GAUCAGCUUCAG*GAA
D-TV1	G*UCGACUAGGACUG*AA
D-TV2	G*CUAGUCGAAGUC*GAA
D-G1A	*A*AGCUGAUCCUGAGAA
D-G1U	*U*AGCUGAUCCUGAGAA
D-G1C	*C*AGCUGAUCCUGAGAA
A-A14G	GGCGAACUCUUCG*G*
A-A14C	GGCGAACUCUUCG*C*
A-A14U	GGCGAACUCUUCG*U*
FH14	GGAUCAGCUCCACGCUGAGUAGACAUACUUGCAAACGCUGCAAACAUAGACGAAGAGUUC

## Supplementary Materials

Figure S1: 9DB1 does not efficiently ligate 5′-AppRNA, Figure S2: Dot plots and predicted secondary structures of SC deoxyribozymes, Figure S3: DNA-catalyzed cleavage of the ligation products reveals 3′-5′-linked phosphodiester bond in the linear RNA product, Figure S4: Synthesis of 2′,5′-branched nucleic acids with 5′-AppRNA and 5′-AppDNA donor oligonucleotides, and Table S1: Mass spectrometry data of synthetic (5′-hexynyl (Hex)-)RNAs and adenylated donor oligonucleotides.
