# Supplementary material for: New Deoxyribozymes for the Native Ligation of RNA
Source: Molecules. 2020 Aug 11;25(16):3650. doi: 10.3390/molecules25163650 (PMC7465978; doi:10.3390/molecules25163650)
Supplement: Supplementary file 1 [file molecules-25-03650-s001.pdf]

Supporting information

New deoxyribozymes for the native ligation of RNA

Carolin P.M. Scheitl, Sandra Lange and Claudia Höbartner*

University of Würzburg, Institute of Organic chemistry, Am Hubland, 97074 Würzburg;

* Correspondence: claudia.hoebartner@uni-wuerzburg.de;

Table of Contents

Supplementary Table 1. Mass spectrometry data of synthetic (5′-hexynyl-)RNAs and adenylated oligonucleotides.

Supplementary Figure 1. 9DB1 does not efficiently ligate 5′App-RNA.

Supplementary Figure 2. Dot plots and predicted secondary structures of SC deoxyribozymes.

Supplementary Figure 3. DNA-catalyzed cleavage of the ligation products reveals 3′-5′-linked phosphodiester bond in the linear RNA product.

Supplementary Figure 4. Synthesis of 2′,5′-branched nucleic acids with 5′-App-RNA and 5′-App-DNA donor oligonucleotides.

Supplementary Table 1. Mass spectrometry data of synthetic (5′-hexynyl (Hex)-)RNAs and adenylated donor oligonucleotides.

Name	5′-sequence-3′	Mass calculated	Mass found
R1 Donor	App-GAGCUGAUCCUGAGAA	5553.4	5553.3
R2 Acceptor	Hex-GGCGAACUCUUCGA	4606.8	4606.4
D-TM	App-GGAUCAGCUUCAGGAA	5553.4	5552.7
D-TV2	App-GCUAGUCGAAGUCGAA	5553.4	5553.4
D-TV1	App-GUCGACUAGGACUGAA	5553.4	5553.3
D-G1U	App-UAGCUGAUCCUGAGAA	5514.3	5514.0
D-G1A	App-AAGCUGAUCCUGAGAA	5537.4	5536.9
D-G1C	App-CAGCUGAUCCUGAGAA	5513.3	5512.8
A-A14G	Hex-GGCGAACUCUUCGG	4622.9	4621.4
A-A14C	Hex-GGCGAACUCUUCGC	4582.8	4581.0
A-A14U	Hex-GGCGAACUCUUCGU	4583.8	4582.0
Donor DNA	App-GAGCTGATCCTGAGAA	5336.9	5336.9
pppRNA	ppp-GAGCUGAUCCUGAGAA	5384.1	5384.6

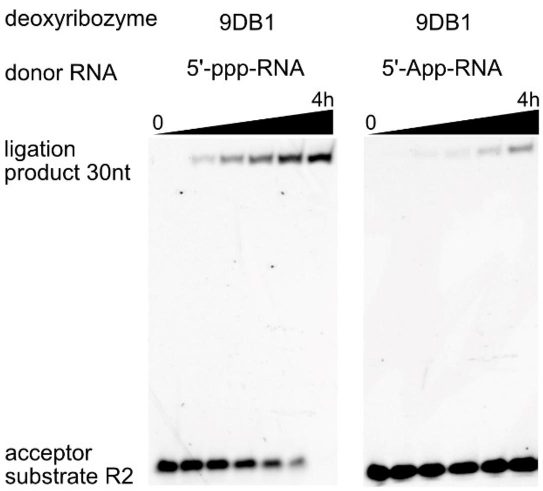

Supplementary Figure 1. 9DB1 does not efficiently ligate 5′App-RNA. Reactions were performed with 40 mM $MnCl_2$, pH 7.5, 37°C, timepoints: 0, 10, 30, 60, 120, 240 min.

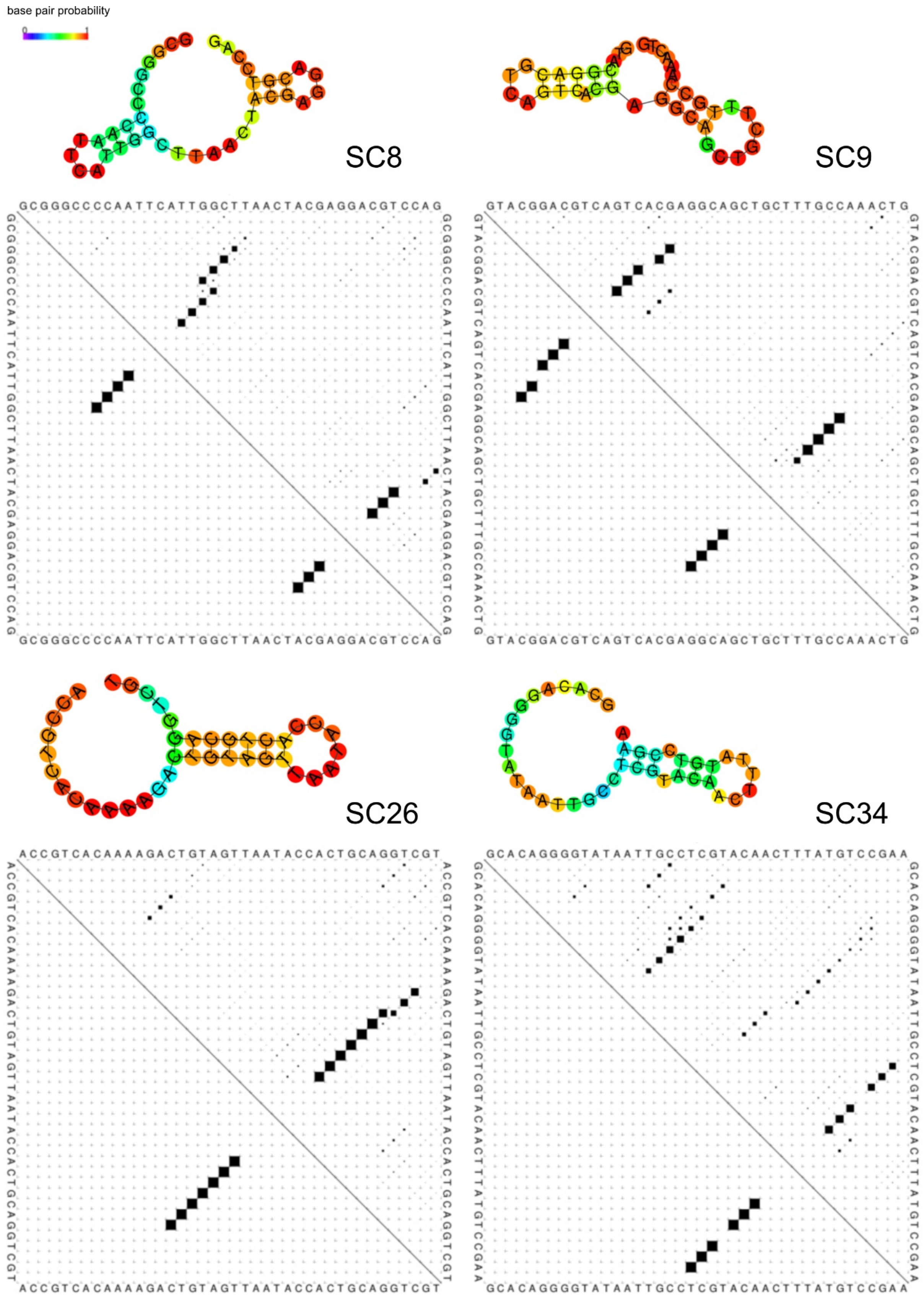

Supplementary Figure 2. Dot plots and predicted minimum free energy (MFE) secondary structures of SC8, SC9, SC26 and SC34 deoxyribozymes, generated by Vienna RNAWebSuite RNA fold http://rna.tbi.univie.ac.at. The MFE structures are colored by base-pairing probabilities; for unpaired regions the color denotes the probability of being unpaired.

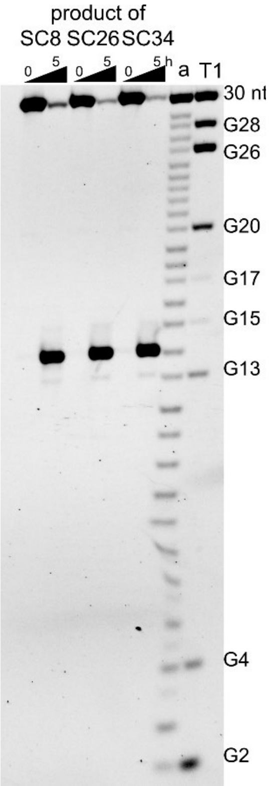

Supplementary Figure 3. The ligation products produced by the SC deoxyribozymes were isolated by PAGE, and individually subjected to cleavage by 8-17 deoxyribozyme D4. The results confirm that the phosphodiester bond produced in the ligation product is of the desired 3′-5′-linked nature. Incubation with 8-17 was carried out with 20 mM Mg^{2+}, 20 mM Mn^{2+} in 40 mM Tris.HCl, 150 mM NaCl, pH 7.5, 37°C, 5 h. The reference lanes for assignment of the cleavage product show alkaline hydrolysis (a) and RNase T1 digestion (T1) products with single-nucleotide resolution.

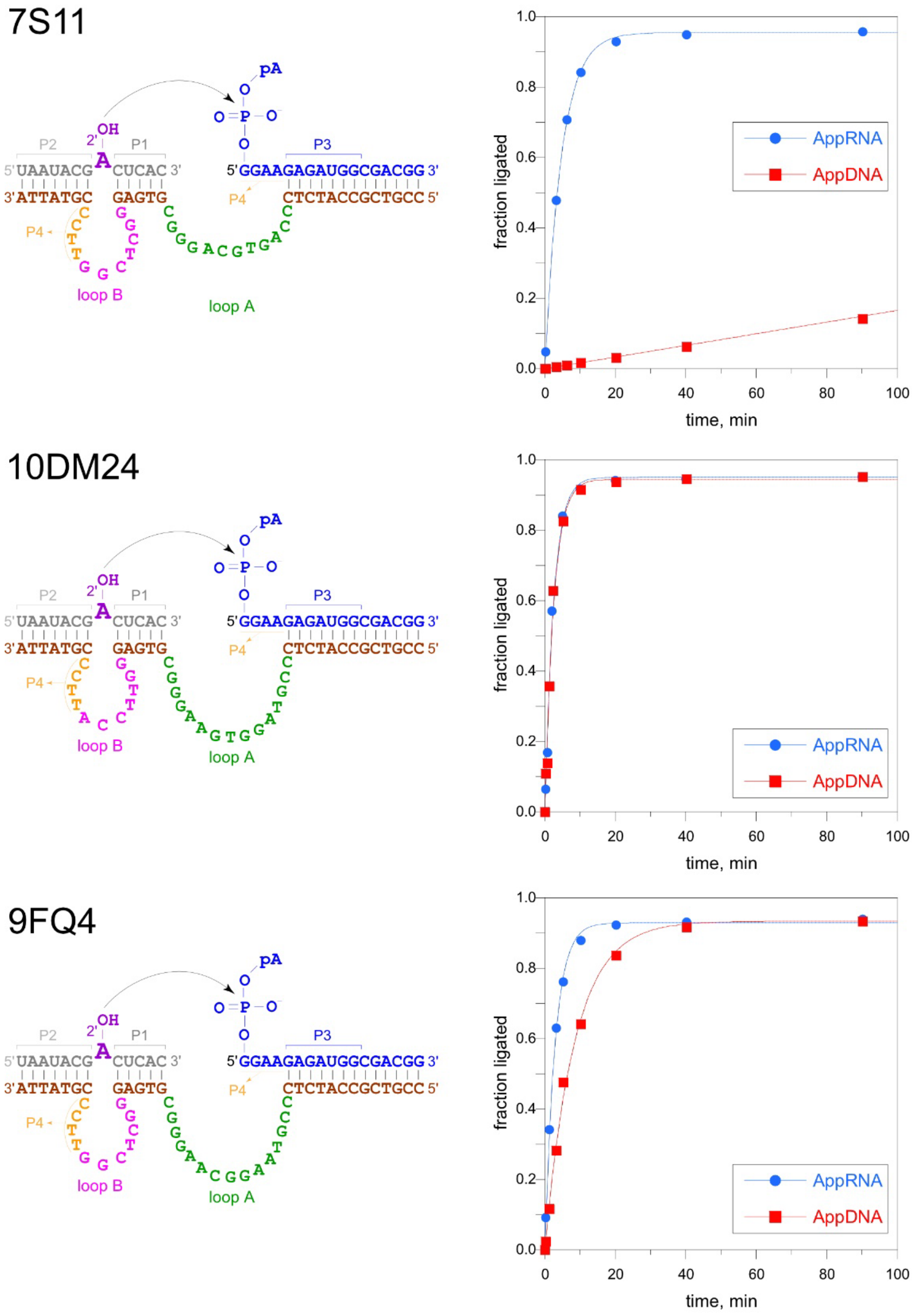

Supplementary Figure 4. Synthesis of 2′,5′-branched nucleic acids with 5′-App-RNA and 5′-App-DNA donor oligonucleotides. Ligation reactions were performed in 40 mM CHES buffer, pH 9.0, 40 mM $MgCl_2$, 37°C. Data courtesy: Falk Wachowius (PhD 2012), AK Höbartner @ MPIbpc, Göttingen.
